# Work-related Musculoskeletal Disorder: An Occupational Disorder of the Goldsmiths in India

**DOI:** 10.4103/0970-0218.66890

**Published:** 2010-04

**Authors:** Tirthankar Ghosh, Banibrata Das, Somnath Gangopadhyay

**Affiliations:** 1 Department of Physiology, Manipal College of Medical Sciences, Pokhara, Nepal; 2 Department of Physiology, South Calcutta Girls College, University of Calcutta, Kolkata; 3 Occupational Ergonomics Laboratory, Department of Physiology, University of Calcutta, Kolkata, WestBengal, India

**Keywords:** Goldsmith, workstation design, musculoskeletal disorders, posture

## Abstract

**Background:**

Gold ornament making industries are one of the widespread small-scale industries of India. These industries belong to the unorganized sector of the state. A large number of goldsmiths are working there for prolonged period in cross leg posture at semi-confined workstation.

**Objectives:**

The aim of this study is to identify Occupational Disorder of the Goldsmiths in India.

**Materials and Methods:**

In the spresent study, 120 male goldsmiths were randomly selected from the Davangere district of Karnataka. A detailed questionnaire study on discomfort feeling was done by the modified Nordic questionnaire, which considering the information about work nature, job stress and discomfort feeling. The existing workstations were assessed by the measurement of work areas. Analysis of body posture by rapid upper limb assessment was done to evaluate the work stress during their job.

**Results:**

From the analysis, it was revealed that MSDs were the major problem of the goldsmiths. The activities of the goldsmiths were also highly repetitive. Moreover, the questionnaire study revealed that most of the workers were affected by occupational disorder like pain at neck (80%), shoulder (20%), wrist (45%), and low back (75%) and also eye problem like irritation (30%) and burning sensation (70%). They also perform their job in hazardous postures. It was recorded that the workstations were poorly illuminated (19 Lux) in respect to precision work. Accidents like cut and burn occurred frequently due to the unsafe condition of the workstation.

**Conclusions:**

From the observation and analysis of the result it was concluded that health of the goldsmiths were highly affected improper body posture and workload. Twisting, bending, and over-reaching are the resultant of poorly designed workstation. These actions force them into a non-neutral position that increases the overall discomfort and pain at the lower back, neck, and shoulders. Moreover, lack of proper illumination at work site also exerts an additional adverse effect on the health of the goldsmiths.

## Introduction

**Figure 1:**
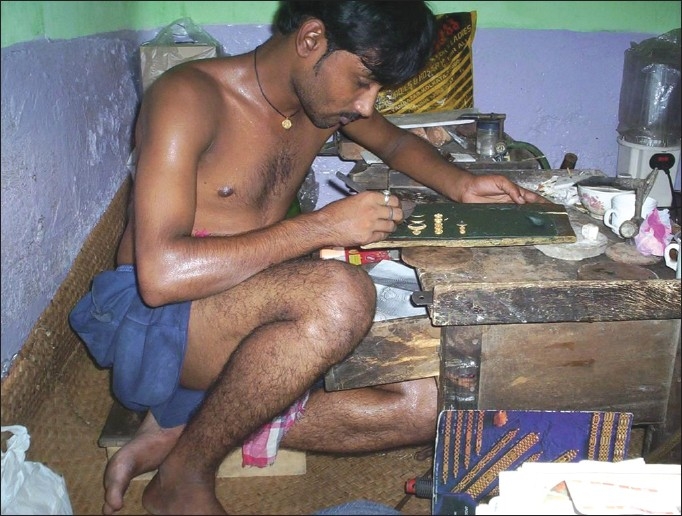
Goldsmiths at their workplace

Gold ornament making industries are one of the widespread small-scale industries of India. These industries belong to the unorganized sector of the state. A large number of goldsmiths are working there for prolonged period in cross leg posture at a semi-confined workstation [Figure 1]. This may have lead to the development of different kinds of musculoskeletal disorders (MSDs) among them (Gangopadhyay *et al*.)(1) (Ijadunola *et al*.)(2) observed that poorly designed workstation promote unnecessary physical efforts, which reduce efficiency and productivity also. Sustaining any static posture, such as sitting, increases the demand on the muscles, ligaments, and other soft tissues of the musculoskeletal system. It is not surprising then that overall discomfort and pain in the back, neck, and shoulders are common symptoms reported by workers who sit for most of their workday. Sitting alters the normal curvature of the spine and puts pressure on the discs. With prolonged sitting this pressure can cause compression of the discs. These resulting chronic back pain and possible nerve damage can impact on workers ability. According to Kapandji (1974),(3) degeneration of the cervical spine, sometimes known as cervical spondylitis, can have serious consequences. Compression of the spinal cord at the level of cervical spine can take place, resulting in weakness and wasting of the upper limbs. This may then spread to the lower limbs. In a previous survey,(4) it was shown that high percentages were suffered from MSDs commonly associated with poor ergonomic design in the workplace. In recent years some workers, trade unions, employers, manufacturers, and researchers have begun to give attention to how workplace design that can improve the health of workers. Ijadunola *et al*.(2) observed that poorly designed workstation promote unnecessary physical efforts, which reduce efficiency and productivity also. Thompson *et al*.(4) show that high percentages were suffered from MSDs commonly associated with poor ergonomic design in the workplace. Without the application of ergonomic principles, tools, machines, equipment, and workstations are often designed without much consideration of the fact that people are of all different heights, shapes, and sizes and have different levels of strength. It is important to consider these differences in order to protect worker’s health and comfort. Without the application of ergonomic principles, workers are often forced to adapt themselves to poor working conditions. Huang and Feuerstein(5) suggested that job redesign and interventions that address a worker’s work style when faced with increased work demands may help reduce the likelihood of musculoskeletal symptoms and/or their intensity. This present study was undertaken to assess the work-related upper body MSDs of the goldsmiths in India.

## Materials and Methods

### Selection of subjects

In this study, the experiment was performed on 120 male goldsmiths. To avoid selection bias, all the workers were selected randomly from the Davangere district of Karnataka.

### Questionnaire study

The modified Nordic questionnaire(6) was applied which included questions emphasizing individual details, type of work, musculoskeletal disorder symptoms, history of accidents, etc. to investigate the discomfort of work. In our present study, this type of questionnaire was applied to evaluate the general physical activity, task variety, work stress, working environment, and workstation design of the goldsmiths.

### Workstation assessment

Workstation of the goldsmiths were observed by measuring the eye distance from the work surface, work surface height from floor, position and source of illumination, working methods, and working tools. The illumination level of the different areas in the three workshops is also measured. The illumination level is measured by the help of Lux-meter (Lutron). The instrument digitally shows the illumination level of that place where the collector is placed.

### Analysis of working posture

The maintenance of posture and the support of load are particular examples of static work. To analyze posture, measurement of the angles between the body parts, distribution of masses of body parts, the forces exerted on the environment during the posture, the length of the time during which specific posture is held, and the effect on the person should be taken into account. A procedure was developed by McAtamney and Corlett(7) to assess the exposure of people to postures, forces, and muscle activities known to contribute to upper limb disorders (ULD).

This rapid upper limb assessment (RULA) technique was used here to assess the postural discomfort of the goldsmith at their average working posture. This was carried out with the aid of digital photography. Later on, stick diagrams were drawn from freeze frame video records and eventually subjected to analysis.

## Results

### Analysis of general information

From the analysis of the questionnaire, results are developed in tabular forms. Table 1 represents the general physical information of the workers. The mean age of the workers are 31 years and they have average 161.2 cms height and 56.9 kgs weight. The years of experience of the workers were calculated from questionnaire and the average goldsmiths are having 16.3 years of experience. The daily work schedule including the mean duration of work and rest as well as the number of working days in a week are also represented in Table 1. It is observed that the goldsmiths work 6 days in a week. The average duration of work par day is 12 h that varies on the demand of work and they work for 6 days in a week.

**Table 1:** General information of the workers

Variables	Mean	SD
Age (years)	31	± 6.38
Height (cm)	161.12	± 11.23
Weight (kg)	56.9	± 9.90
Years of experience	16.3	± 7.22
Duration of work per day (in hour)	12	± 2.11
Duration of rest per day (in hour)	1	± 1.00
Number of working days in a week	6	

### Analysis of occupational disorders

During questionnaire study 80.0% of the goldsmiths were reported the feeling of discomfort, among them 70% were feeling discomfort during work and 30% feeling discomfort during rest. The feeling of discomfort in different body parts of the workers is shown inTable 2. It is observed that the feelings of discomfort among the goldsmiths were mainly related to MSDs like pain at neck (80%), low back (75%), wrist (45%), shoulder (20%), and also eye problem like irritation (30%) and burning sensation (70%) [Tables3 and 4].

**Table 2:** Average information of the existing workstation

Readings	Eye distance from work surface (cm)	Work surface height from floor (cm)	Illumination level of work surface (lLx)	Light arrangements
Mean	19 cm.	37 cm.	19 Lux	Four tubes
SD	± 1.71	± 4.52	± 2.10	(160 watt)

**Table 3:** Discomfort feeling during work and during rest inthe goldsmiths

Goldsmiths	Discomfort feeling	Discomfort feeling during work	Discomfort feeling during rest
	96(80)	84(70)	36(30)

Figures in parenthesis are percentages

**Table 4:** Discomfort feeling at different body parts

Body parts	Neck	Shoulder	Wrist	Low back	Eye (irritation)	Eye (burning sensation)
Number of effected	96(80)	24(20)	54(45)	90(75)	36(30)	84(70)

Figures in parenthesis are percentages

### Workstation assessment

**Figure 2:**
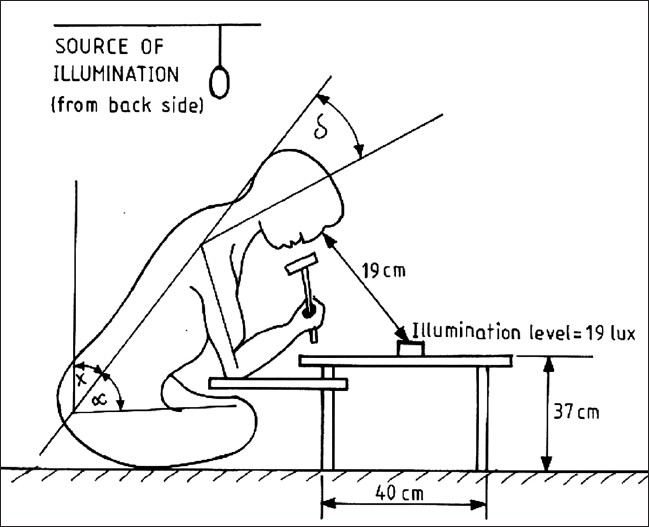
Goldsmiths at their average workstation

From the workstation assessment, it was found that [Table 2 and Figure 2], in average 10-11 goldsmiths are worked in 180 cms/280 cms room which are poorly illuminated (19 Lux) also. Often, when performing the precise job, the workers lean forward to obtain good vision of the work area. Goldsmiths work for prolonged time with neck inclination (41° ) and back inclination (52° ) (forward) from the vertical sitting posture for:

1. The sitting placement is too stretched from the work surface,
2. Work surface is too low, and
3. The visual demands of the task requiring a specific eye location (They are maintaining average 19 cms distance between eye and work surface).

### Analysis of working posture

The average working postures of the goldsmiths at their working condition (cross-legged) were analyzed by the RULA method [Table 5]. The analysis revealed that the posture requires investigation and changes immediately. This indicates that the workers adopt awkward posture at their daily work process.

**Table 5:** Analysis of working posture of the goldsmiths (by the RULA method)

Posture	RULA score	Action level
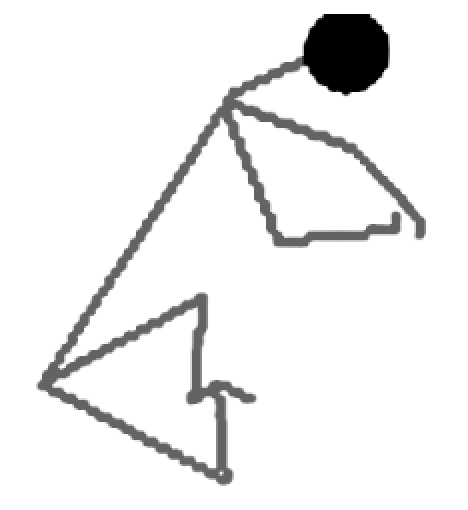	7	Investigation and changes required immediately
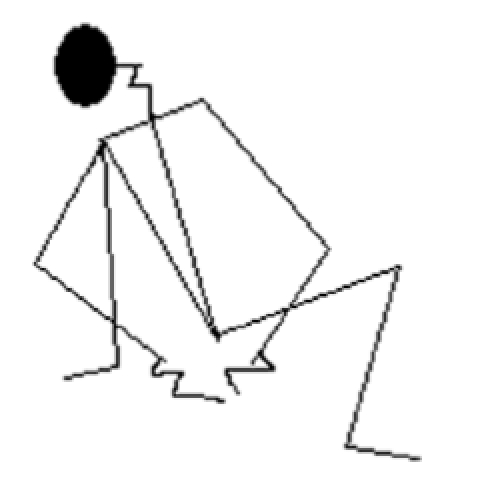	7	Investigation and changes required immediately
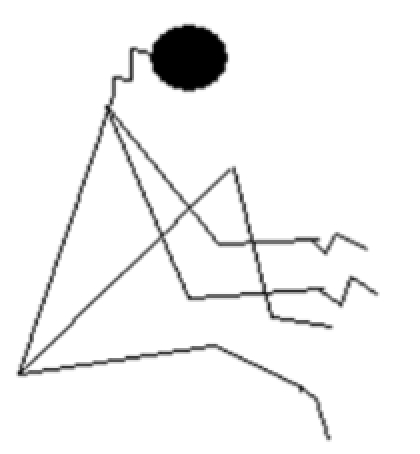	6	Investigate further and change soon

## Discussion

The results of this study revealed that the goldsmiths are engaged in prolonged forward bending posture in there working condition. The amount and quality of forward-bent posture and the techniques of work influence the compressive force on the vertebral discs and the electromyography of erector spine muscles (Chaffin and Anderson).(8) Studies of Jonsson *et al*.,(9) Kilbom *et al*.(10), Kilbom and Persson,(11) dealt with the same cohort; female electronics workers followed for 3 successive years. These studies found significant association between posture variables and neck MSDs. Similarly, this study finds that 80.0% of the goldsmiths were feeling discomfort. It also observed that the feelings of discomfort among the goldsmiths were related to MSDs like pain at neck (80%) and low back (75%). This finding also corroborates with the analysis of cross-legged posture of the goldsmiths by the RULA method and reveals that the posture requires investigation and changes immediately. This indicates that the workers by adopting awkward posture at work, most often suffer from MSDs particularly affecting the low back and neck region.

Forces acting on the spine in forward inclined posture are conceder as cervical extensor muscle force and lumber extensor muscle force. Kumar and Scaife(12) proved that the cervical extensor muscle force and lumber extensor muscle force are found from moment equations which primarily depend on the size of the angels of “δ” neck inclination and “α” back inclination from the vertical sitting posture. Thus, for even a 30° inclination angle from the vertical, the moment and corresponding muscle forces values are at 50% of the values achieved at 90° (horizontal). The proportion of neck strength in respect of neck angle (Snyder *et al*.)(13) shows that we have average neck strength at 30° neck inclination. A study by Jorgensen(14) revealed that most of the men are capable of maintaining a 20° forward bending posture, because the load-moment increases rapidly for each degree of back inclination above 20°. It is also recognized that when work surface is too low, a person not only leans forward, but also may lower and rotate the shoulders forward causing fatigue and pain in the levator scapulae muscles (Cailliet(15). From the observation and analysis of the existing posture of the goldsmiths, it was found that they have to stay for prolonged period with having neck inclination (41° ) and back inclination (52°) (forward) from the vertical sitting posture due to their poor workstation design. Ankrum and Nemeth(16) suggest that adopting postures involving relatively extended head or neck for prolonged periods are likely to lead to musculoskeletal discomfort.

The permissible range of illuminance, recommended by the Illuminating Engineering Society (IES, 1981),(17) should be 300 Lux for fine precision work, like the work of the goldsmiths. On comparing this value with the observed illuminance level in the goldsmiths workshops, it was observed that the levels were very low than the recommended value (RQQ, 1980).(18) This may be the prime cause for the visual problem among the goldsmiths. From the questionnaire analysis, it was found that 57% of the goldsmiths agreed that there exists rigidity in work methods and procedures. This does not allow them the flexibility that is essential for the successful completion of any type of job. 64% reported that supervisory pressure is high and 82% reported that work demand target-specific productivity. This suggests that they had to work under stressed condition also. From the questioner study it was also found that the goldsmiths are affected by eye burning sensation (70%), red eyes (85%), blurred vision (40%), and headaches (52%) during work [Table 6].

**Table 6:** Visual discomfort feeling during work in the goldsmiths

Goldsmiths	Eye burning	Red eyes	Blurred vision	Headaches
	84(70)	102(85)	48(40)	60(52)

Figures in parenthesis are percentages

## Conclusion

From the observation and analysis of the result it can be concluded that the goldsmiths are working in awkward postures, with the potential risks of MSDs primarily affecting the low-back and neck region. This can be attributed to the improper design of the workstation. Twisting, bending, and over-reaching are the resultant of poorly designed workstation. These actions force the spine into a non-neutral position that increases the overall discomfort and pain particularly at the lower back, neck, and shoulders, which indicate that the goldsmiths are/may be affected by work-related upper body MSDs. Moreover, they have to work for a prolong period of time remaining in such constrained and awkward postures, which further amplifies their discomfort feeling. The working environment also affects them to a great extent. Lack of proper illumination at work site exerts an additional adverse effect on the eyes. Thus this study indicates the appalling condition of the goldsmiths.

To overcome such problem, the existing posture can be eliminated by ergonomically modified work desk. This modification may increase the safety of the workers at the work. Recommendations like changing the work height by using a proper work desk was provided to improve work condition by avoiding the forward bending posture. Also to improve the workstation design the goldsmith should be provided proper illumination to their work site, which can reduce the visual discomfort to some extent. Finally, since prolonged sitting in cross leg posture is clearly an additional risk factor affecting the musculoskeletal system in these setting, goldsmith should be strongly suggested to take rest pauses during work period.
